# Assessment of Leisure Time Physical Activity and Brain Health in a Multiethnic Cohort of Older Adults

**DOI:** 10.1001/jamanetworkopen.2020.26506

**Published:** 2020-11-19

**Authors:** Yian Gu, Juliet M. Beato, Erica Amarante, Anthony G. Chesebro, Jennifer J. Manly, Nicole Schupf, Richard P. Mayeux, Adam M. Brickman

**Affiliations:** 1 Taub Institute for Research in Alzheimer’s Disease and the Aging Brain, Columbia University, New York, New York; 2 Department of Neurology, Columbia University, New York, New York; 3 Gertrude H. Sergievsky Center, Columbia University, New York, New York; 4 Joseph P. Mailman School of Public Health, Department of Epidemiology, Columbia University, New York, New York

## Abstract

**Importance:**

Results from longitudinal studies suggest that regular leisure time physical activity (LTPA) is associated with reduced risk of dementia or Alzheimer disease. Data on the association between LTPA and brain magnetic resonance imaging (MRI) measures remain scarce and inconsistent.

**Objective:**

To examine the association of LTPA and MRI-assessed brain aging measures in a multiethnic elderly population.

**Design, Setting, and Participants:**

This cross-sectional study included 1443 older (≥65 years) adults without dementia who were participants of the Washington/Hamilton Heights-Inwood Columbia Aging Project study. LTPA, from self-reported questionnaire, was calculated as metabolic equivalent of energy expenditure. Both moderate to vigorous LTPA, assessed as meeting Physical Activity Guidelines for Americans (≥150 minutes/week) or not, and light-intensity LTPA were also examined.

**Exposures:**

LTPA.

**Main Outcomes and Measures:**

Primary outcomes included total brain volume (TBV), cortical thickness, and white matter hyperintensity volume, all derived from MRI scans with established methods and adjusted for intracranial volume when necessary. We examined the association of LTPA with these imaging markers using regression models adjusted for demographic, clinical, and vascular risk factors.

**Results:**

The 1443 participants of the study had a mean (SD) age of 77.2 (6.4) years; 921 (63.8%) were women; 27.0%, 34.4%, and 36.3% were non-Hispanic White, non-Hispanic African American, and Hispanic individuals, respectively; and 27.3% carried the apolipoprotein E (*APOE*) ɛ4 allele. Compared with the LTPA of nonactive older adults, those with the most LTPA had larger (in cm^3^) TBV (β [SE], 13.17 [4.42] cm^3^; *P* = .003; *P* for trend = .006) and greater cortical thickness (β [SE], 0.016 [0.008] mm; *P* = .05; *P* for trend = .03). The effect size comparing the highest LTPA level with the nonactive group was equivalent to approximately 3 to 4 years of aging (β for 1 year older, −3.06 and −0.005 for TBV and cortical thickness, respectively). A dose-response association was found and even the lowest LTPA level had benefits (eg, TBV: β [SE], 9.03 [4.26] cm^3^; *P* = .03) compared with the nonactive group. Meeting Physical Activity Guidelines for Americans (TBV: β [SE], 18.82 [5.14] cm^3^; *P* < .001) and light-intensity LTPA (TBV: β [SE], 9.26 [4.29] cm^3^; *P* = .03) were also associated with larger brain measures. The association between LTPA and TBV was moderated by race/ethnicity, sex, and *APOE* status, but generally existed in all subgroups. The results remained similar after excluding participants with mild cognitive impairment.

**Conclusions and Relevance:**

In this study, more physical activity was associated with larger brain volume in older adults. Longitudinal studies are warranted to explore the potential role of physical activity in brain health among older individuals.

## Key Points

**Question:**

Is physical activity associated with brain volume and white matter hyperintensity burden?

**Findings:**

In this cross-sectional study of 1443 older (≥65 years) individuals without dementia, more physical activity was associated with larger brain volumes.

**Meaning:**

The findings of this study suggest that there may be a potential beneficial role of physical activity on brain health.

## Teaser

This cross-sectional study examines the association between leisure time physical activity and measures of brain health assessed by magnetic resonance imaging in older adults.

## Introduction

A large body of evidence from longitudinal studies^1,2,3,4^ has found that regular leisure time physical activity (LTPA) is associated with reduced risk of dementia or Alzheimer disease (AD). Accordingly, the recently released second edition of Physical Activity Guidelines for Americans (PAGA)^5,6^ added cognitive health and reduced dementia risk to the growing list of LTPA benefits.

Multiple brain structural changes, including both neurodegeneration such as volume loss and cerebrovascular lesions such as white matter hyperintensities (WMH), are powerful predictors for subsequent AD development.^7^ It would therefore be interesting to examine whether LTPA is associated with these brain measures. Several observational and interventional studies have found that greater activities are associated with larger brain volume^8,9,10,11,12,13,14^ and/or less WMH,^15^ but inconsistent results also have been reported.^15,16,17^ Few studies have taken into consideration different activity intensity levels. While PAGA and many previous studies focused on moderate to vigorous LTPA, it is important to evaluate whether light-intensity LTPA can help slow the brain morphological changes among older adults who may have limited-moderate to vigorous LTPA. Similarly, it would be of practical interest to evaluate what would be a threshold level for older adults to gain brain health benefits. In addition, with LTPA as a promising precision prevention target, it is important to evaluate the role of LTPA in brain health among certain subgroup populations, especially those at higher risk of developing AD such as racial/ethnic minority groups, women, and genetic risk factor carriers.

Previous research in the Washington/Hamilton Heights-Inwood Columbia Aging Project (WHICAP)^1,2^ showed that participating more in LTPA was associated with lower AD risk. The aim of this study was to examine whether higher LTPA is associated with larger brain volume, cortical thickness, and less WMH as measured by magnetic resonance imaging (MRI) in this multiethnic elderly cohort.

## Methods

### Participants and Setting

WHICAP is a community-based, longitudinal study on aging and dementia in a multiethnic sample of older (aged ≥65 years) residents of uptown Manhattan.^18^ There were 3 recruitment waves in 1992, 1999, and 2009, all using similar sampling, assessments, and study procedures.^2,18^ Participants repeated the baseline examinations every 18 to 24 months in follow-up appointments. The diagnosis of dementia and the type of dementia were based on standard research criteria.^19,20^ The diagnosis of mild cognitive impairment (MCI) used Petersen^21^ criteria as described elsewhere.^22^

A total of 1584 participants in the WHICAP study received MRI assessment. The detailed information regarding the enrollment into the neuroimaging substudy has been described previously.^7,23,24^ We excluded 63 participants who were diagnosed with dementia around the time of the scan. Among the remaining 1521 participants, LTPA was not available for 78. Compared with the 78 participants with incomplete data, the 1443 participants included in the current study were older (mean [SD], 77.2 [6.4] vs 73.9 [7.2]) but otherwise similar.

Ethical approval was obtained from the institutional review boards of Columbia University. All participants provided written informed consent.

### MRI Protocol

#### Image Acquisition

Scans were acquired on a 1.5T Intera scanner (Philips Healthcare) for the 1999 wave and a 3T Achieva scanner (Philips Healthcare) for the 2009 wave at Columbia University.^7^ For the 1999 wave, T1-weighted (repetition time [RT] = 20 ms, echo time [ET] = 2.1 ms, field of view [FOV] = 240 cm, 256 × 160 matrix, 1.3 mm slice thickness) and T2-weighted fluid-attenuated inversion recovery (FLAIR) (RT = 11 000 ms, ET = 144.0 ms, inversion time = 2800 ms, FOV = 25 cm, 2 excitations, 256 × 192 matrix with 3 mm slice thickness) images were acquired in the axial orientation. For the 2009 wave, T1-weighted (RT = 6.6 ms, ET = 3.0 ms, FOV = 256 × 256 × 165, 1.0 mm slice thickness) and T2-weighted FLAIR (RT = 8000 ms, ET = 332 ms, FOV = 240 × 240 × 180, 0.43 mm slice thickness) images were acquired axially.

#### Volume and Cortical Thickness Measures

All T1 images were analyzed using Freesurfer (versions 5.1 and 6.0 for 1999 and 2009 waves, respectively; Laboratory for Computational Neuroimaging at the Athinoula A. Martinos Center for Biomedical Imaging).^7^ Freesurfer output underwent visual quality control and manual correction whenever necessary.^23^ We examined brain volumetric measures (cm^3^) including total brain volume (TBV), total gray matter volume (TGMV), total white matter volume (TWMV), and hippocampal volume. To adjust for differences in head size across participants, regression models were run with intracranial volume (ICV) as the independent variable and brain volume as the outcome variable, and the regression residuals were then used in the analyses.^25^ We calculated mean cortical thickness (mm)^26^ across all regions of interest within each participant.

#### WMH Quantification

The quantification of global and regional WMH volumes has been previously described.^7,24^ First, each participant’s T2-weighted FLAIR image was skull stripped, and a single gaussian curve was fit to voxel intensity values in the resultant image. An intensity threshold of 1.8 and 2.1 SD above the mean intensity value for 1999 and 2009 waves, respectively, were set to define the lower boundary of hyperintense voxels, and voxels above that threshold were labeled. The resulting map was further visually inspected and corrected for false-positive and false-negative errors for each participant. Total WMH volume in cubic centimeters was defined as the number of labeled voxels multiplied by voxel dimensions. Log-transformed total WMH volume was used in the analysis.

### Leisure Time Physical Activity

Information about current LTPA was collected using the Godin leisure time exercise questionnaire.^27^ Past studies have shown that reports of LTPA using the Godin questionnaire are reliable^27,28^ and valid.^1^ At baseline, participants were queried about the frequency of LTPA during the most recent 2 weeks and duration (measured in metabolic-equivalent minutes [MET-minutes]) per session for 3 different intensity categories of LTPAs: vigorous, moderate, and light.^2^ Total MET-minutes in 2 weeks for each intensity category was calculated,^2,27^ and summed across the 3 categories to obtain each individual’s total LTPA amount (MET-minutes per 2 wk). The LTPA was further categorized into no LTPA and tertiles of nonzero values (low, middle, or high LTPA).

We also determined whether an individual did or did not meet the current PAGA guidelines, ie, averaging 150 minutes/week or more of moderate and/or vigorous LTPA. In addition, an individual was considered as meeting the guidelines if their light-intensity LTPA was above 250 minutes/week (light PAGA), which had an equivalent total LTPA amount as the PAGA (ie, 750 METs-minutes/week). The light LTPA was categorized into no, lower-middle, higher-middle, or meeting light PAGA, with the middle 2 groups based on median split (120 minutes/week).

### Covariates

Information about age, sex, education, ethnicity, body mass index (BMI, calculated as weight in kilograms divided by height in meters squared), smoking status, and alcohol use was obtained from baseline interviews. Race and ethnicity were self-reported using the format of the 2000 US Census. Participants were then assigned to 1 of 3 groups: African American (non-Hispanic), Hispanic, White (non-Hispanic), or other. Years of education was self-reported and used as a continuous variable. Caloric intake was calculated from the baseline food-frequency questionnaire. Apolipoprotein E (*APOE*) genotype was categorized as ε4 carriers (either 1 or 2 ε4) or noncarriers. Presence or absence of heart disease, diabetes, hypertension, head injury, and depression were based on self-reported information as well as the use of medication for any conditions, and stroke was determined by self-report, neurologic examination, or a review of medical records. Alcohol use and smoking history was self-reported by standard questionnaires.^26^ Self-reported occupation was used as a categorical variable (ie, manager or professional vs others).

### Statistical Analysis

Brain measures, LTPA levels, and other characteristics of participants were compared across the levels of LTPAs using ANOVA for continuous variables and χ^2^ test for categorical variables. We used multivariable linear regression models to estimate the association between LTPA and imaging markers (ie, TBV, cortical thickness, WMH volume). The analyses were performed in a series of models, with Model 1 adjusted for age, enrollment wave, and ICV (except for cortical thickness); Model 2 additionally adjusted for demographic and socioeconomic variables including sex, race/ethnicity, education, occupation, and MCI status at scan visit; and Model 3 further adjusted for vascular and other comorbidities including comorbidity score and BMI. We also examined whether meeting the PAGA guideline and light LTPA were associated with brain measures by including them simultaneously in the models, adjusted for the same variables as above.

Effect modifications by sex, race/ethnicity, and *APOE* were tested by including an interaction term (ie, ordinal LTPA x effect modifier; *P* for interaction) into the regression models with TBV as the outcome variable, then adjusting for Model 2 covariates, followed by stratified analyses by significant effect modifiers. To reduce the possibility of potential reverse causality and recall bias, we excluded participants with MCI. Post-hoc analyses were performed, globally and separately for left and right hemispheres, for regional volumes: TGMV, TWMV, and hippocampal volume.

All analyses were performed using SPSS Statistics 25.0 (IBM). The level of statistical significance was set at *P* < .05.

## Results

### Characteristics of the Study Participants

The MRI scans were assessed a mean (SD) 2.69 (2.17) years after the LTPA assessment in the 1443 participants included in the study. Approximately two-thirds of these participants were women (921 [63.8%]), and the mean (SD) age was 77.2 (6.4) years; 390 (27.0%) of participants were non-Hispanic White, 497 (34.4%) were African American, 524 (36.3%) Hispanic, and 32 (2.2%) were of other race/ethnicity; 27.3% carried the apolipoprotein E (*APOE*) ɛ4 allele. (Table 1).

**Table 1.** Characteristics of Study Participants According to the Total Physical Activity Amount

Characteristics	Participants, No. (%)					*P* value
	LTPA^a^				All (N = 1443)	
	None (n = 322)	Low (n = 355)	Middle (n = 393)	High (n = 373)		
Women^b^	221 (68.6)	239 (67.3)	233 (59.3)	228 (61.1)	921 (63.8)	.02
Age at baseline, mean (SD), y	78.7 (6.6)	78.4 (6.2)	76.9 (6.4)	75.1 (5.7)	77.2 (6.4)	<.001
Race/ethnicity^b^						
Non-Hispanic						<.001
White	60 (18.6)	87 (24.5)	115 (29.3)	128 (34.3)	390 (27.0)	
African American	101 (31.4)	134 (37.7)	139 (35.4)	123 (33.0)	497 (34.4)	
Hispanic	157 (48.8)	128 (36.1)	126 (32.1)	113 (30.3)	524 (36.3)	
Other	4 (1.2)	6 (1.7)	13 (3.3)	9 (2.4)	32 (2.2)	
Education, mean (SD), y	10.1 (4.9)	11.4 (4.7)	11.9 (4.6)	13 (4.5)	11.6 (4.8)	<.001
Apolipoprotein ε4 carrier^b^	71 (22.0)	98 (27.6)	113 (28.8)	112 (30.0)	394 (27.3)	.14
MCI	75 (23.3)	55 (15.5)	84 (21.4)	57 (15.3)	271 (18.8)	.01
Cognitive *z* score, mean (SD)^c^	0.15 (0.61)	0.35 (0.57)	0.41 (0.57)	0.61 (0.54)	0.39 (0.59)	<.001
BMI, mean (SD)^d^	30.1 (10.9)	28.6 (7.9)	28 (7.6)	27.4 (5)	28.5 (8.1)	<.001
Diabetes	114 (35.4)	104 (29.3)	118 (30.0)	80 (20.4)	416 (28.8)	.001
Heart disease	141 (43.8)	158 (44.5)	142 (36.1)	118 (31.6)	559 (38.7)	.001
Hypertension	279 (86.6)	301 (84.8)	312 (79.4)	275 (73.7)	1167 (80.9)	<.001
Stroke	59 (18.3)	59 (16.6)	49 (12.5)	31 (8.3)	198 (13.7)	<.001
Ever smoked	131 (40.7)	156 (43.9)	165 (42.0)	180 (48.3)	632 (43.8)	.19
Ever use alcohol^e^	54 (16.8)	90 (25.4)	112 (28.5)	104 (27.9)	360 (24.9)	.001
Caloric intake, mean (SD), kcal	1369 (516)	1388 (485)	1393 (491)	1450 (474)	1401 (492)	.21
Duration from LTPA to MRI scan, mean (SD), y	2.82 (2.13)	2.88 (2.05)	2.77 (2.22)	2.30 (2.22)	2.69 (2.17)	.001
Total LTPA, mean (SD) [range], MET-min/2 wk	0	448 (235) [6-840]	1319 (312) [900-2040]	4647 (4496) [2050-45 960]	1671 (2927) [0-45 960]	<.001
LTPA						
Vigorous	0	4 (50)	75 (279)	1235 (3203)	340 (1716)	<.001
Moderate	0	28 (111)	152 (393)	777 (1508)	250 (857)	<.001
Light	0	417 (247)	1094 (474)	2648 (2935)	1098 (1824)	<.001
LTPA guideline						
Meeting PAGA (moderate or vigorous LTPA ≥150 min/wk)	0	0	12 (3.1)	124 (32.5)	136 (9.4)	<.001
Meeting light PAGA (light LTPA ≥250 min/wk)	0	0	56 (14.2)	265 (71.2)	321 (22.6)	
Higher-middle light LTPA (light LTPA 120 to <250 min/wk)	0	33 (9.3)	269 (68.4)	58 (15.6)	360 (25.3)	
Lower-middle light LTPA (light LTPA >0 and <120 min/wk)	0	306 (86.4)	45 (11.5)	30 (8.1)	381 (26.8)	
No light LTPA	304 (100)	15 (4.2)	23 (5.9)	19 (5.1)	361 (25.4)	
MRI measures, mean (SD), cm^3^						
ICV	1331 (164)	1349 (161)	1360 (183)	1395 (178)	1360 (174)	<.001
TBV	860 (92)	874 (88)	882 (101)	900 (106)	880 (98)	<.001
TGMV	519 (50)	528 (47)	539 (57)	549 (55)	534 (54)	<.001
TWMV	378 (53)	388 (51)	392 (55)	405 (56)	391 (55)	<.001
Hippocampal volume	6.76 (0.94)	6.93 (0.84)	7.04 (0.89)	7.12 (0.9)	6.97 (0.90)	<.001
Cortical thickness, mean (SD), mm	2.45 (0.12)	2.45 (0.11)	2.46 (0.11)	2.47 (0.11)	2.46 (0.11)	.004
Log-transformed WMH, mean (SD), cm^3^	0.23 (0.63)	0.33 (0.63)	0.27 (0.63)	0.32 (0.63)	0.23 (0.63)	.13

Abbreviations: BMI, body mass index; ICV, intracranial volume; LTPA, leisure time physical activity; MET, metabolic equivalent; MCI, mild cognitive impairment; MRI, magnetic resonance imaging; PAGA, Physical Activity Guidelines for Americans; TBV, total brain volume; TGMV, total gray matter volume; TWMV, total white matter volume; WMH, white matter hyperintensities.

^a^ The cutoffs for the low-middle and middle-high LTPA were 900 and 2050 MET-minutes/2 wk respectively, equivalent to approximately weekly 0.8 h vigorous, 1.5 h moderate, or 2.5 h light LTPA, and weekly 1.9 h vigorous, 3.4 h moderate, or 5.7 h light LTPA for meeting middle and high LTPA cutoffs, respectively.

^b^ Percentages may not equal 100% due to rounding. One participant was missing information on *APOE* ε4; 58 participants were missing information on BMI; and 1251 participants were missing caloric intake information.

^c^ Cognitive *z *score was calculated from a neuropsychological battery.^23^

^d^ Calculated as weight in kilograms divided by height in meters squared.

^e^ Based on self-reported answers of having 1 or more alcoholic drinks per week.

Those who had more LTPA were younger (eg, mean [SD] age: low LTPA, 78.4 [6.2] years vs high LTPA, 75.1 [5.7] years; *P* < .001), more likely to be men, more likely to be White and less likely to be Hispanic, less likely to have MCI, and had more years of education, lower BMI, fewer comorbidities, and larger brain volumes (Table 1). There was no difference across LTPA levels in *APOE* ε4 allele status or WMH volume (Table 1).

In this study population of older adults, most participants performed some amount of light LTPA, but only 16.7% performed moderate and 10.3% performed vigorous LTPA. Overall, 136 (9.4%) of the participants met the PAGA.

### Association Between LTPA and Brain Measures

More LTPA was associated with larger TBV and more cortical thickness, both with a dose-response association (Model 2: TBV, *P* for trend = .006; cortical thickness, *P* for trend = .03; Table 2; Figure). Compared with no LTPA in Model 2, the highest level of LTPA had a β (SE) of 13.17 (4.42) cm^3^ larger size in TBV (*P* = .003; *P* for trend = .006) and a 0.016 (0.008) mm (*P* = .053; *P* for trend = .03) larger size in cortical thickness, respectively (Table 2), which is the equivalent to approximately 3 to 4 years of aging (β = −3.06 cm^3^ on TBV and −0.005 mm on cortical thickness for 1-year increase in age). When additionally adjusted for BMI and comorbidities in Model 3, the associations were attenuated generally but remained significant for TBV (β [SE], 9.47 [4.51] cm^3^) (Table 2). Meeting PAGA was associated with larger TBV (β [SE], 18.82 [5.14]) and more cortical thickness (β [SE], 0.02 [0.01]) (Table 3). Meeting light PAGA was also associated with larger TBV (β [SE], 9.26 [4.29]) (Model 2 in Table 3) independent of moderate or vigorous LTPA, although the magnitude of association was about half of the size of meeting PAGA through moderate to vigorous LTPA

**Table 2.** Association Between Leisure Time Physical Activity (LTPA) Levels and Brain Magnetic Resonance Imaging (MRI) Measures

Brain measures	LTPA levels^a^	Model 1^b^		Model 2^c^		Model 3^d^	
		β (SE)	*P* value	β (SE)	*P* value	β (SE)	*P* value
TBV	None	1 [Reference]	NA	1 [Reference]	NA	1 [Reference]	NA
	Low	9.778 (4.214)	.02	9.030 (4.262)	.03	7.418 (4.312)	.09
	Middle	11.703 (4.145)	.01	8.853 (4.218)	.04	6.588 (4.286)	.12
	High	15.647 (4.313)	<.001	13.166 (4.423)	.003	9.467 (4.505)	.04
	*P* value for trend	NA	<.001	NA	.006	NA	.06
Cortical thickness	None	1 [Reference]	NA	1 [Reference]	NA	1 [Reference]	NA
	Low	0.003 (0.008	.71	0.004 (0.008)	.65	0.004 (0.008)	.60
	Middle	0.011 (0.008)	.16	0.013 (0.008)	.10	0.010 (0.008)	.25
	High	0.018 (0.008)	.04	0.016 (0.008)	.05	0.014 (0.009)	.11
	*P* value for trend	NA	.02	NA	.03	NA	.09
WMH	None	1 [Reference]	NA	1 [Reference]	NA	1 [Reference]	NA
	Low	0.066 (0.045)	.15	0.081 (0.046)	.08	0.101 (0.047)	.03
	Middle	−0.001 (0.045)	.99	0.028 (0.045)	.54	0.034 (0.047)	.47
	High	0.004 (0.046)	.94	0.041 (0.048)	.39	0.055 (0.049)	.26
	*P* value for trend	NA	.67	NA	.69	NA	.59
TGMV	None	1 [Reference]	NA	1 [Reference]	NA	1 [Reference]	NA
	Low	6.143 (2.596)	.02	4.756 (2.621)	.07	4.365 (2.622)	.10
	Middle	11.039 (2.553)	<.001	8.513 (2.594)	.001	7.629 (2.606)	.003
	High	9.778 (2.657)	<.001	7.204 (2.720)	.008	4.834 (2.739)	.08
	*P* value for trend	NA	<.001	NA	.004	NA	.045
TWMV	None	1 [Reference]	NA	1 [Reference]	NA	1 [Reference]	NA
	Low	5.588 (2.816)	.05	5.931 (2.892)	.04	4.930 (2.939)	.09
	Middle	5.289 (2.770)	.06	4.974 (2.862)	.08	3.819 (2.921)	.19
	High	7.510 (2.882)	.01	7.509 (3.001)	.01	5.349 (3.071)	.08
	*P* value for trend	NA	.02	NA	.03	NA	.14
Hippocampal volume	None	1 [Reference]	NA	1 [Reference]	NA	1 [Reference]	NA
	Low	0.130 (0.056)	.02	0.127 (0.058)	.03	0.129 (0.059)	.03
	Middle	0.142 (0.056)	.01	0.143 (0.057)	.01	0.154 (0.059)	.01
	High	0.059 (0.058)	.30	0.066 (0.060)	.27	0.071 (0.062)	.25
	*P* value for trend	NA	.32	NA	.29	NA	.26

Abbreviations: NA, not applicable; TBV, total brain volume; TGMV, total gray matter volume; TWMV, total white matter volume; WMH, white matter hyperintensity.

^a^ The 4 levels of total LTPA were high (≥2050 MET-min/2 wk), middle (900 to <2050 MET-min/2 wk), low (0 to <900 MET-min/2 wk), and none.

^b^ Model 1 adjusted for age at baseline, intracranial volume, and wave.

^c^ Model 2 adjusted for sex, race/ethnicity, education, mild cognitive impairment status, occupation, and apolipoprotein E ε4.

^d^ Model 3 adjusted for body mass index, and comorbidities including psychiatric diseases, diabetes, insulin treatment, heart disease, hypertension, head injury, depression, stroke, smoking, and heavy alcohol use.

**Figure.**
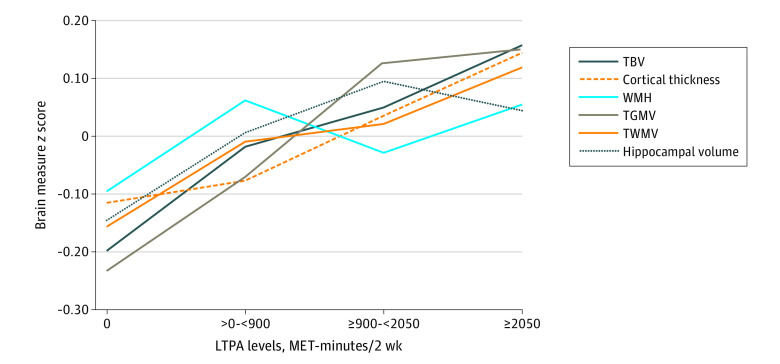
Brain Measures by Leisure Time Physical Activity Levels Brain measures are standardized (by *z* scores) for convenient comparisons across different brain measures. Solid lines represent significant associations between leisure time physical activity (LTPA) and brain measures (see trend test in Table 2), while dotted and dashed lines indicate nonsignificant associations. TBV indicates total brain volume; TGMV, total gray matter volume; TWMV, total white matter volume; WMH, white matter hypersensitivity.

**Table 3.** Association Between Leisure Time Physical Activity Levels According to Physical Activity Guidelines for Americans (PAGA) and Brain Magnetic Resonance Imaging Measures

LTPA^a^	Model 1^b^		Model 2^c^		Model 3^d^			
	β (SE)	*P* value	β (SE)	*P* value	β (SE)			*P* value
**TBV**								
LTPA levels								
Not meeting PAGA	[Reference]	NA	[Reference]	NA	[Reference]			NA
Meeting PAGA	20.724 (5.115)	<.001	18.815 (5.141)	<.001	16.967 (5.175)			.001
Light LTPA								
None	[Reference]	NA	[Reference]	NA	[Reference]			NA
Lower-middle light LTPA	10.220 (4.030)	.01	9.599 (4.072)	.02	7.516 (4.116)			.07
Higher-middle light LTPA	9.542 (4.104)	.02	8.091 (4.153)	.05	6.333 (4.228)			.13
Meet light PAGA	9.984 (4.248)	.02	9.262 (4.294)	.03	6.287 (4.344)			.15
* P* value for trend	NA	.03	NA	.05	NA			.20
**Cortical thickness**								
LTPA levels								
Not meeting PAGA	[Reference]	NA	[Reference]	NA	[Reference]			NA
Meeting PAGA	0.022 (0.010)	.03	0.020 (0.010)	.04	0.017 (0.010)			.08
Light LTPA								
None	[Reference]	NA	[Reference]	NA	[Reference]			NA
Lower-middle light LTPA	0.005 (0.008)	.52	0.005 (0.008)	.53	0.004 (0.008)			.61
Higher-middle light LTPA	0.011 (0.008)	.16	0.012 (0.008)	.13	0.011 (0.008)			.16
Meet light PAGA	0.014 (0.008)	.09	0.012 (0.008)	.14	0.009 (0.008)			.28
* P* value for trend	NA	.06	NA	.08	NA			.19
**WMH**								
LTPA levels								
Not meeting PAGA	[Reference]	NA	[Reference]	NA	[Reference]			NA
Meeting PAGA	0.045 (0.055)	.42	0.071 (0.056)	.20	0.088 (0.057)			.12
Light LTPA								
None	[Reference]	NA	[Reference]	NA	[Reference]			NA
Lower-middle light LTPA	0.062 (0.043)	.15	0.081 (0.044)	.07	0.090 (0.045)			.05
Higher-middle light LTPA	−0.024 (0.044)	.59	0.005 (0.045)	.91	0.010 (0.046)			.83
Meet light PAGA	0.018 (0.046)	.70	0.050 (0.046)	.28	0.058 (0.047)			.22
* P* value for trend	NA	.80	NA	.63	NA			.55
**TGMV**								
LTPA levels								
Not meeting PAGA	[Reference]	NA	[Reference]	NA	[Reference]		NA	
Meeting PAGA	12.168 (3.151)	<.001	10.190 (3.163)	.001	9.098 (3.148)		.004	
Light LTPA								
None	[Reference]	NA	[Reference]	NA	[Reference]		NA	
Lower-middle light LTPA	6.355 (2.483)	.01	5.142 (2.505)	.04	4.856 (2.504)		.05	
Higher-middle light LTPA	8.876 (2.529)	<.001	7.054 (2.555)	.006	6.512 (2.572)		.01	
Meet light PAGA	6.670 (2.617)	.01	5.559 (2.642)	.04	3.650 (2.642)		.17	
* P* value for trend	NA	.005	NA	.02	NA		.21	
**TWMV**								
LTPA levels								
Not meeting PAGA	[Reference]	NA	[Reference]	NA	[Reference]	NA		
Meeting PAGA	9.263 (3.405)	.006	9.560 (3.473)	.005	8.617 (3.510)	.01		
Light LTPA								
None	[Reference]	NA	[Reference]	NA	[Reference]	NA		
Lower-middle light LTPA	5.799 (2.683)	.03	5.894 (2.751)	.03	5.145 (2.792)	.07		
Higher-middle light LTPA	4.127 (2.733)	.13	4.161 (2.805)	.14	3.145 (2.868)	.27		
Meet light PAGA	4.804 (2.828)	.09	4.750 (2.901)	.10	3.225 (2.946)	.27		
* P* value for trend	NA	.15	NA	.17	NA	.41		
**Hippocampal volume**								
LTPA levels								
Not meeting PAGA	[Reference]	NA	[Reference]	NA	[Reference]	NA		
Meeting PAGA	0.138 (0.069)	.04	0.158 (0.069)	.02	0.160 (0.071)	.02		
Light LTPA								
None	[Reference]	NA	[Reference]	NA	[Reference]	NA		
Lower-middle light LTPA	0.143 (0.054)	.008	0.142 (0.055)	.01	0.148 (0.056)	.008		
Higher-middle light LTPA	0.120 (0.055)	.03	0.118 (0.056)	.04	0.125 (0.058)	.03		
Meet light PAGA	0.056 (0.057)	.32	0.052 (0.058)	.37	0.060 (0.059)	.31		
* P* value for trend	NA	.38	NA	.44	NA	.39		

Abbreviations: LTPA, leisure time physical activity; NA, not applicable; PAGA, Physical Activity Guidelines for Americans; TBV, total brain volume; TGMV, total gray matter volume; TWMV, total white matter volume; WMH, white matter hyperintensity.

^a^ Two levels of PAGA, PAGA met and not met, indicated 150 min/wk (approximately 750 METs/wk) or more and less than 150 min/wk of moderate or vigorous LTPA, respectively. Four levels of total light LTPA were light PAGA met, higher-middle, lower-middle, and none light LTPA, indicating ≥250 min/wk (approximately 750 METs/wk), 120-250 min/wk, >0 to 120 min/wk, and no light LTPA, respectively.

^b^ Model 1 adjusted for age at baseline, intracranial volume, and recruitment wave.

^c^ Model 2 adjusted for sex, race/ethnicity, education, mild cognitive impairment status, occupation, and apolipoprotein E ε4.

^d^ Model 3 adjusted for body mass index and comorbidities including psychiatric diseases, diabetes, insulin treatment, heart disease, hypertension, stroke, head injury, depression, smoking, and heavy alcohol use.

 In general, there was no association between LTPA and WMH. After adjusting for BMI and comorbidities, low levels of LTPA were associated with larger WMH volume (eg, β [SE], 0.10 [0.05]; see Model 3 in Tables 2 and 3).

### Supplementary Analyses

The association between LTPA with TBV was modified by race/ethnicity such that it was stronger for non-Hispanic White individuals than for Hispanic individuals (*P* for interaction = .05); the difference from non-Hispanic African American individuals was not significant (*P* for interaction = .07) (Table 4). However, benefits of LTPA were seen in all racial/ethnic groups, depending on amount and intensity-type of LTPA (Table 4).

**Table 4.** Association of Leisure Time Physical Activity and Total Brain Volume, by Race/Ethnicity, Sex, and Apolipoprotein E ɛ4^a^

Characteristic		LTPA					PAGA		Light LTPA				
		None	Low (>0 to <450 METs/wk)	Middle (450 to <1025 METs/wk)	High (≥1025 METs/wk)	*P* value for trend	Not meeting PAGA	Meeting PAGA	No LTPA	Lower-middle light LTPA (>0 to <120 min/wk)	Higher-middle light LTPA (120 to <250 min/wk)	Light LTPA meeting PAGA (≥250 min/wk)	*P* value for trend
**Race/ethnicity**													
Non-Hispanic White and other	No.	63	88	124	130	NA	341	59	83	104	115	98	NA
	β (SE)	1 [Reference]	−1.591 (8.904)	5.245 (8.434)	17.621 (8.805)	NA	1 [Reference]	13.072 (7.88)	1 [Reference]	12.367 (7.986)	0.678 (7.764)	22.225 (8.131)	NA
	*P* value	NA	.86	.53	.05	.02	NA	0.10	NA	.12	.93	.006	.04
Non-Hispanic African American	No.	92	132	137	118	NA	414	58	109	143	133	87	NA
	β (SE)	1 [Reference]	10.798 (7.798)	10.486 (7.796)	12.526 (8.351)	NA	1 [Reference]	19.045 (8.34)	1 [Reference]	2.195 (7.358)	7.955 (7.479)	4.684 (8.336)	NA
	*P* value		.17	.18	.13	.18	NA	.02	NA	.77	.29	.57	.39
Hispanic	No.	149	126	121	109	NA	480	17	150	124	102	121	NA
	β (SE)	1 [Reference]	11.814 (6.081)	8.075 (6.154)	4.57 (6.462)	NA	1 [Reference]	39.395 (12.826)	1 [Reference]	12.246 (6.033)	13.848 (6.365)	−1.32 (6.178)	NA
	*P* value	NA	.05	.19	.48	.49	NA	.002	NA	.04	.03	.83	.95
**Sex**													
Men	No.	95	110	158	138	NA	425	57	111	120	147	114	NA
	β (SE)	1 [Reference]	2.011 (7.933)	4.366 (7.398)	18.644 (7.857)	NA	1 [Reference]	22.749 (8.256)	1 [Reference]	7.298 (7.536)	6.072 (7.183)	10.493 (7.57)	NA
	*P* value	NA	.80	.56	.02	.02	NA	.006	NA	.33	.40	.17	.21
Women	No.	209	236	224	219	NA	800	77	231	251	203	192	NA
	β (SE)	1 [Reference]	13.437 (4.949)	12.277 (5.070)	9.121 (5.269)	NA	1 [Reference]	15.050 (6.612)	1 [Reference]	11.333 (4.775)	9.832 (5.047)	8.488 (5.151)	NA
	*P* value	NA	.007	.02	.08	.12	NA	.02	NA	.02	.05	.10	.13
***APOE* status**													
*APOE* ɛ4 noncarrier	No.	236	248	270	249	NA	901	88	257	265	248	219	NA
	β (SE)	1 [Reference]	6.915 (4.958)	10.084 (4.902)	16.419 (5.169)	NA	1 [Reference]	20.348 (6.366)	1 [Reference]	9.655 (4.784)	9.113 (4.880)	12.038 (5.060)	NA
	*P* value	NA	.16	.04	.001	.001	NA	.001	NA	.04	.06	.02	.02
*APOE* ɛ4 carrier	No.	68	98	112	108	NA	334	46	85	106	102	87	NA
	β (SE)	1 [Reference]	13.838 (8.373)	4.456 (8.291)	4.698 (8.689)	NA	1 [Reference]	15.309 (8.906)	1 [Reference]	9.447 (7.807)	3.356 (7.954)	1.701 (8.261)	NA
	*P* value	NA	.10	.59	.59	.93	NA	.09	NA	.23	.67	.84	.92

Abbreviations: *APOE*, apolipoprotein ɛ4; LTPA, leisure time physical activity; NA, not applicable; PAGA, Physical Activity Guidelines for Americans.

^a^ Regression models were adjusted for Model 2 covariates including age at baseline, ICV (except for cortical thickness), recruitment wave, sex, race/ethnicity, education, mild cognitive impairment status, occupation, and *APOE* ε4, except for the variable tested as an effect moderator.

Sex (*P* for interaction = .05) and *APOE* genotype (*P* for interaction = .09) also modified the interaction between LTPA and TBV, although the difference was not significant. Meeting PAGA was associated with TBV in both men and women (Table 4). In *APOE* ε4 noncarriers, the results were similar to the main analyses, while in *APOE* ε4 carriers, only low LTPA or meeting PAGA were associated with larger TBV (Table 4).

In 1172 participants who did not have MCI, LTPA remained to be associated with all brain volumes and cortical thickness, but not with WMH. Meeting PAGA also remained to be significantly associated with TBV (data not shown).

Higher LTPA (Table 2) and meeting PAGA and performing light LTPA were associated with larger TGMV, TWMV and hippocampal volume (Table 3). Physical activity was positively associated with both left and right hemisphere cortical gray matter, cortical white matter, and hippocampal volumes (eTable in the Supplement). Middle LTPA (β [SE], 0.246 [0.086]; *P* = .004) and higher-middle light LTPA (β [SE], 0.223 [0.091]; *P* = .01) were associated with hippocampal volume in Hispanic individuals but not in non-Hispanic White or African American individuals; similar results for low LTPA were not significant (β [SE], 0.166 [0.085]; *P* = .05). Both LTPA (comparing high vs non: β [SE], 0.210 [0.109]; *P* for trend = .03) and light-intensity LTPA (light PAGA vs no light PAGA: β [SE], 0.203 [0.104]; *P* for trend = .03) were associated with hippocampal volume in men, but total LTPA was not significantly associated with hippocampal volume in women. In *APOE* ɛ4 noncarriers, the results were similar to the main analyses (data not shown), while in *APOE* ɛ4 carriers, performing low (β [SE], 0.281 [0.113]; *P* = .01) or middle (β [SE], 0.264 [0.112]; *P* = .02) LTPA was associated with larger hippocampal volume.

## Discussion

In the present study of older adults, we found individuals who reported more LTPA had larger brain volume and cortical thickness than those who reported less LTPA. Our findings are in line with previous reports that show positive associations between LTPA and brain volume among older adults.^8,9,10,11,12,29,30,31^ Only 2 previous studies^9,31^ examined different LTPA amount levels in relation to brain volume, but no dose-response relationship was established. We found a dose-response association between total LTPA and brain volume or cortical thickness, with benefit gain starting from even low amounts of LTPA. The Framingham study^31^ found light-intensity PA, but not meeting PAGA from moderate or vigorous activities, was associated with higher TBV. In contrast, in a study of 323 older participants with MCI,^13^ moderate and vigorous, but not light-intensity, LTPA was associated with less brain atrophy. We found both light and moderate or vigorous intensity activities were independently associated with larger brain volumes, albeit with different effect sizes. Considering the much larger prevalence of light activities in the older adult population than moderate or vigorous activities, future health education needs to take into consideration both the effect size and practical feasibility for a better physical activity promotion in elderly populations.

For individuals who are at higher risk of developing AD, it is particularly important to identify potential protective factors that can slow down the trajectory into the clinical stage of the disease. To our knowledge, this is the first study to show the association between LTPA and brain volume among African American and Hispanic individuals who had a higher risk of AD than White individuals.^18^ We also found a significant association between LTPA and brain volume in both women and men, although to a lesser extent for women than for men. Our findings are in accordance with a 2018 longitudinal study^32^ that found a significant association of LTPA with brain atrophy in men but not in women, and may help explain why older women have cognitive benefits from exercise to a lesser extent than older men.^33,34^ However, other studies found association between LTPA and hippocampal volumes among older women but not older men.^35,36,37^ The discrepancy might be due to the difference in LTPA level definition and brain regions examined.^37^ While the association of LTPA with TBV among *APOE* ɛ4 carriers was not as strong as among noncarriers, a low to middle level of LTPA was significantly associated with larger hippocampal volume in carriers. Previous studies have found either no interaction^38^ between LTPA and *APOE* status on cognition or that *APOE* ε4 carriers benefitted more from the exercise intervention than *APOE* ε4 noncarriers.^39^ Thus, the current study adds to the literature suggesting that LTPA might be an important intervention target for brain health and dementia prevention among *APOE* ε4 carriers. Among specific brain regions, hippocampal volume seems to be the key region responsive to LTPA,^9,30,37,40,41^ probably due to its plasticity and susceptibility to age-related atrophy.^42^ Thus, among at-risk individuals carrying the *APOE* ɛ4 allele, the association between LTPA and hippocampal volume may indeed reflect the results of this susceptible brain region being compensated by LTPA.

We found LTPA in general was not associated with WMH. According to a 2016 review,^15^ most studies did not find a negative association between LTPA and WMH. We found that compared with no activity, low LTPA, particularly of light intensity, was associated with larger WMH volumes, echoing the findings of the Framingham Heart Study.^31^ The reason for this seemingly counterintuitive finding is unclear. With no clear dose-response association and with little evidence, cautions are needed to interpret the results. Nevertheless, future studies could elucidate the potential association between LTPA and WMH, especially with consideration of cognitive-stimulating leisure activities, light-intensity LTPA, and vascular comorbidities.

The exact mechanisms for the positive association between LTPA and brain volume remains to be clarified but likely involves multiple biological mechanisms. Higher levels of physical activity are associated with higher levels of the neurotrophins such as brain-derived neurotrophic factor,^8^ synaptic plasticity,^43^ increased cerebral blood flow,^44^ and decreased β-amyloid 1-42 in cerebrospinal fluid.^45^ In addition to direct effects, physical activity may also contribute to brain maintenance, such as reducing the aging effect on amyloid deposition or glucose metabolism,^46^ and brain resilience, such as reducing the effect of amyloid on TBV.^47^

### Limitations and Strengths 

There are a few limitations in our study. This is a cross-sectional study, so we could not rule out the reverse causality. Self-reported activities may have certain misclassification errors which might have biased our results toward null. However, the design of the questionnaire allows analysis of LTPA intensity levels and reflects long-term habitual physical activity. While we adjusted for many potential confounders, residual confounding from other factors, such as diet^26^ and sleep,^48^ might remain. The subgroup analyses were limited by small sample size and might have been underpowered. We did not examine particular types of LTPA, but it might be less important than amount of activities.^49^

There are many strengths of this study. Our study is among the largest ones that have examined LTPA and brain measures in community-based populations. The study controlled for many potential confounders including demographics, occupation, and comorbidities. We examined both the total amount/volume and intensity of LTPA and found even low-dose and low-intensity activities might have benefits for brain health. We found certain effect modifications by race/ethnicity, sex, and *APOE* ɛ4 status, supporting future investigation among at risk subpopulations. We found significant associations between LTPA and brain volume in 3 racial/ethnic groups, thus increasing the generalizability to the increasingly diverse US population.

## Conclusions

Habitual LTPA is associated with larger brain volumes in older adults. Future longitudinal studies are warranted to confirm whether LTPA can prevent brain atrophy in older individuals.

## Notes

Supplement. **eTable.** Association of LTPA and Brain Volumes in Left and Right Hemispheres
