# Supplementary material for: Assessment of Leisure Time Physical Activity and Brain Health in a Multiethnic Cohort of Older Adults
Source: JAMA Netw Open. 2020 Nov 19;3(11):e2026506. doi: 10.1001/jamanetworkopen.2020.26506 (PMC7677758; doi:10.1001/jamanetworkopen.2020.26506)
Supplement: Supplement. — eTable. Association of LTPA and Brain Volumes in Left and Right Hemispheres [file jamanetwopen-e2026506-s001.pdf]

Supplemental Online Content

Gu Y, Beato JM, Amarante E, et al. Assessment of leisure time physical activity and brain health in a multiethnic cohort of older adults. *JAMA Netw Open*. 2020;3(11):e2026506. doi:10.1001/jamanetworkopen.2020.26506

eTable. Association of LTPA and Brain Volumes in Left and Right Hemispheres

This supplemental material has been provided by the authors to give readers additional information about their work.

© 2020 Gu Y et al. *JAMA Network Open.*

eTable. Association of LTPA and brain volumes in left and right hemispheres.

	LTPA	Total				LTPA	Left hemisphere			Right hemisphere		
	**Level**	**B**	**SE**	**P**		**Level**	**B**	**SE**	**P**	**B**	**SE**	**P**
TGMV	High	**7.204**	**2.720**	**0.008**	Cortex GMV	High	**3.104**	**1.139**	**0.006**	2.118	1.113	0.06
	Middle	**8.513**	**2.594**	**0.001**		Middle	**2.346**	**1.086**	**0.03**	1.768	1.061	0.10
	Low	4.756	2.621	0.07		Low	1.911	1.097	0.08	1.390	1.072	0.20
	None	ref		/		None	ref			ref		
	p-trend			**0.004**		p-trend			**0.008**			0.06
TWMV	High	**7.509**	**3.001**	**0.01**	Cortex WMV	High	**3.496**	**1.604**	**0.03**	2.647	1.578	0.09
	Middle	4.974	2.862	0.08		Middle	2.255	1.530	0.14	1.411	1.505	0.35
	Low	**5.931**	**2.892**	**0.04**		Low	2.459	1.545	0.11	2.095	1.521	0.17
	None	ref		/		None						
	p-trend			**0.03**		p-trend			**0.05**			0.16
Hippocampal volume	High	0.066	0.060	0.27	Hippocampal volume	High	0.037	0.034	0.28	0.031	0.032	0.35
	Middle	**0.143**	**0.057**	**0.01**		Middle	0.061	0.033	0.06	**0.083**	**0.031**	**0.008**
	Low	**0.127**	**0.058**	**0.03**		Low	0.054	0.033	0.10	**0.074**	**0.031**	**0.02**
	None	ref		/		None	ref			ref		
	p-trend			0.29		p-trend			0.30			0.37
TGMV	Meeting PAGA	**9.098**	**3.148**	***0.004***	Cortex GMV	Meeting PAGA	**4.780**	**1.860**	**0.010**	**4.381**	**1.825**	**0.02**
	Not meeting PAGA	ref		/		Not meeting PAGA	ref.			ref		
	Light-PAGA	3.650	2.642	*0.17*		Light-PAGA	2.327	1.553	0.13	1.377	1.525	0.37
	Higher-middle light LTPA	**6.512**	**2.572**	***0.01***		Higher-middle light LTPA	2.141	1.502	0.15	0.944	1.475	0.52
	Lower-middle light LTPA	**4.856**	**2.504**	***0.05***		Lower-middle light LTPA	2.607	1.473	0.08	2.099	1.446	0.15
	None	ref		/		None	ref.			ref		
	P-trend			*0.11*		P-trend			0.18			0.54
TWMV	Meeting PAGA	**8.617**	**3.510**	***0.01***	Cortex WMV	Meeting PAGA	**4.401**	**1.322**	**0.001**	**4.102**	**1.292**	**0.002**
	Not meeting PAGA	ref		/		Not meeting PAGA	ref.			ref		
	Light-PAGA	3.225	2.946	*0.27*		Light-PAGA	**2.509**	**1.104**	**0.02**	1.383	1.079	0.20
	Higher-middle light LTPA	3.145	2.868	*0.27*		Higher-middle light LTPA	2.028	1.067	0.06	1.683	1.044	0.11
	Lower-middle light LTPA	5.145	2.792	*0.07*		Lower-middle light LTPA	1.950	1.047	0.06	1.648	1.024	0.11
	None	ref		/		None	ref.			ref		
	P-trend			*0.42*		P-trend			**0.03**			0.21
Hippocampal volume	Meeting PAGA	**0.160**	**0.071**	***0.02***	Hippocampal volume	Meeting PAGA	**0.088**	**0.040**	**0.03**	0.069	0.038	0.07
	Not meeting PAGA	ref		/		Not meeting PAGA	ref.			ref		
	Light-PAGA	0.060	0.059	*0.31*		Light-PAGA	0.033	0.033	0.33	0.021	0.032	0.50
	Higher-middle light LTPA	**0.125**	**0.058**	***0.03***		Higher-middle light LTPA	**0.064**	**0.032**	**0.05**	0.055	0.030	0.07
	Lower-middle light LTPA	**0.148**	**0.056**	***0.01***		Lower-middle light LTPA	**0.061**	**0.032**	**0.05**	**0.082**	**0.030**	**0.006**
	None	ref		/		None	ref.			ref		
	P-trend			*0.42*		P-trend			0.31			0.69

Results were from linear regression model adjusted for age at baseline, ICV, wave, sex, race/ethnicity, education, MCI status, occupation, and apolipoprotein E ε4 allele. All statistical significance ($p \leq 0.05$) results are shown in bold. Four levels of total LTPA were high, middle, low, and none, indicating activities of ≥2050, 900-2050, >0-900, and zero MET-minutes/2-week, respectively. LTPA (leisure time physical activity), TBV (total brain

© 2020 Gu Y et al. *JAMA Network Open.*

volume), TGMV (total gray matter volume), TWMV (total white matter volume). Two levels of PAGA, PAGA met and not met, indicating 150 min/wk (approximately 750 METs/wk) or more, and less than 150 min/wk of moderate or vigorous LTPA, respectively. Four levels of total Light-LTPA were light-PAGA met, higher-middle, lower-mid, and none light-LTPA, indicating ≥250 min/wk (approximately 750 METs/wk), 120-250 min/wk, >0 - 120 min/wk, and no light-LTPA, respectively.

© 2020 Gu Y et al. *JAMA Network Open.*
